# Caffeoylquinic Acid Derivatives Extract of* Erigeron multiradiatus* Alleviated Acute Myocardial Ischemia Reperfusion Injury in Rats through Inhibiting NF-KappaB and JNK Activations

**DOI:** 10.1155/2016/7961940

**Published:** 2016-07-19

**Authors:** Zhifeng Zhang, Yuan Liu, Xuecong Ren, Hua Zhou, Kaishun Wang, Hao Zhang, Pei Luo

**Affiliations:** ^1^Institute of Qinghai-Tibetan Plateau, Southwest University for Nationalities, Chengdu, Sichuan 610041, China; ^2^State Key Laboratories for Quality Research in Chinese Medicines, Macau University of Science and Technology, Macau; ^3^Department of Medicinal Natural Products, West China School of Pharmacy, Sichuan University, Chengdu, Sichuan 610041, China

## Abstract

*Erigeron multiradiatus* (Lindl.) Benth. has been used in Tibet folk medicine to treat various inflammatory diseases. The aim of this study was to investigate antimyocardial ischemia and reperfusion (I/R) injury effect of caffeoylquinic acids derivatives of* E. multiradiatus* (AE)* in vivo* and to explain underling mechanism. AE was prepared using the whole plant of* E. multiradiatus* and contents of 6 caffeoylquinic acids determined through HPLC analysis. Myocardial I/R was induced by left anterior descending coronary artery occlusion for 30 minutes followed by 24 hours of reperfusion in rats. AE administration (10, 20, and 40 mg/kg) inhibited I/R-induced injury as indicated by decreasing myocardial infarct size, reducing of CK and LDH activities, and preventing ST-segment depression in dose-dependent manner. AE decreased cardiac tissue levels of proinflammatory factors TNF-*α* and IL-6 and attenuated leukocytes infiltration. AE was further demonstrated to significantly inhibit I-*κ*B degradation, nuclear translocation of p-65 and phosphorylation of JNK. Our results suggested that cardioprotective effect of AE could be due to suppressing myocardial inflammatory response and blocking NF-*κ*B and JNK activation pathway. Thus, caffeoylquinic acids might be the active compounds in* E. multiradiatus* on myocardial ischemia and be a potential natural drug for treating myocardial I/R injury.

## 1. Introduction

Accumulating clinical and experimental evidences showed that myocardial infarction (MI) was associated with a strong inflammatory response [1–3]. Injury was more dominant from reperfusion than that from ischemia and release of inflammatory substance was thought to be the main cause of reperfusion-associated pathologies, such as cardiomyocyte death, contraction band necrosis, no reflow, and ventricular arrhythmia [4–6]. MI triggers a reparative response in which 3 overlapping phases (inflammatory, proliferative, and remodeling phase) are well described by Turillazzi et al. [7]. Following cardiomyocyte death, an intense inflammatory response is detectable in the infarcted myocardium. Inhibition of myocardial inflammatory response might have promise as a therapeutic strategy for cardiac repair. Initially, studies on modulation of myocardial inflammatory response were focused on effector mediators such as polymorphonuclear neutrophil infiltration, and macrophage migration. Experimental studies in animals also indicated that therapeutic interventions by anti-inflammatory agents reduced infarct size and attenuated cardiac dysfunction. Even though numerous studies have been dedicated to identify agents from natural herb medicine modulating the inflammatory response after MI, no promising data from clinical trials have been observed so far.

Caffeoylquinic acid (CQ), containing quinic acid as the parent moiety and phenylpropanoids such as caffeic acid as the substituted moiety, has a wide range of pharmacological actions [8]. Limited number of studies addressing the mechanism by which CQs exerted its anti-inflammatory effects has focused on the immunomodulatory effect or peroxynitrite-scavenging action. However, there is no report on the inhibition of CQs against the inflammatory consequences of I/R in heart.

*Erigeron multiradiatus* (Lindl.) Benth., a kind of biennial or perennial herb, is mainly distributed in Qinhai-Tibet plateau of China at altitudes ranging from 2600 to 4300 m [9]. In traditional Tibetan medicine,* E. multiradiatus* has been used for years to treat various diseases, including hyperpiesia, enteritis, diarrhea, and food poisoning as well as fever and cough. Studies of photochemistry reported that flavonoids, phenolic acids, and sterols were isolated and identified in* E. multiradiatus* [10, 11]. The crude extract of* E. multiradiatus* has been reported to be having important pharmacological actions such as anti-inflammatory, hepatoprotective, and antidiabetic effects in our previous studies [12, 13]. Furthermore, we have been interested in the CQs from* E. multiradiatus* because of its high abundance. The primary CQs in* E. multiradiatus* are dicaffeoylquinic acids and the content was 81.7% by UV spectrophotometer. Despite the fact that crude extract of* E. multiradiatus* exerted anti-inflammatory effects, till now there is no any report on the effects of CQs from* E. multiradiatus* on reducing myocardial injury induced by I/R. Here, we extracted CQs from* E. multiradiatus* and assessed whether CQs have a beneficial effect against I/R-induced myocardial injury by inhibition of inflammatory response. Using* in vivo* model of I/R, we examined possible mechanisms involved in NF-KappaB and JNK activations pathways (Figure 8).

## 2. Materials and Methods

### 2.1. Drugs and Reagents

Standards of 1,3-dicaffeoylquinic acid, 3,4-dicaffeoylquinic acid, 3,5-dicaffeoylquinic acid, 4,5-dicaffeoylquinic acid, 3,4,5-tricaffeoylquinic acid, and Erigoster B (purity > 98%) were purchased from National Institute for Food and Drug Control (NIFDC, Beijing, China). Acetonitrile was HPLC grade and deionized water was prepared using a Millipore water purification system. Caffeic acid phenethyl ester (CAPE) was from Sigma-Aldrich (purity > 97%). Polyclonal rabbit anti-mouse phosphor-NF-*κ*B, I*κ*B-*α*, and phosphor-JNK antibodies were purchased from Cell Signaling Technology or BD Transduction Laboratories. 2,3,5-Triphenyltetrazolium chloride (TTC) were purchased from Sigma (St. Louis, MO, USA). Creatine kinase-MB (CK-MB) and lactate dehydrogenase (LDH) kits were purchased from STANBIO (Texas, USA).

### 2.2. Animals

Adult male Sprague-Dawley rats weighing 220–280 g were purchased from the Animal House, Pharmacy Discipline, Sichuan University (Chengdu, China). The animals were fed individually under constant temperature (25 ± 1°C) and humidity with a 12 h light/dark cycle and with a rodent standard diet with free access to water ad libitum. Animal care and treatment procedures were in accordance with the Institutional Guidelines and Animal Ordinance, which was approved by the local Animal Ethics Committees of the Faculty of Medicine, Sichuan University.

### 2.3. Preparation of Caffeoylquinic Acid Derivatives Extract (AE)

The whole plant of* Erigeron multiradiatus* (family: Asteraceae) was collected in Ganzi (Sichuan Province, China) and identified by Hao Zhang, Professor of Taxonomy and Pharmaceutical Botany in Pharmacy School of West China, Sichuan University (Chengdu, Sichuan Province, China). The voucher specimen (E12025) was deposited in the herbarium of Pharmacy School of West China, Sichuan University. The dried herb of* E. multiradiatus* (2.0 kg) was ground to powder, reflux-extracted with 75% ethanol for three times (12 liters for 2 hours each time), and centrifuged through membrane filtration. The filtrate solution was evaporated to obtain (396.5 g) a crude extraction. The crude extract was enriched by macroporous resin. The resin column was successively eluted by water, 40% ethanol, and 60% ethanol. Then, the 60% ethanol elution was collected and concentrated under the vacuum to remove ethanol. The residual solution was extracted for three times by acetic ether. Then, the acetic ether solution was concentrated and dried to obtain AE (7.2 g).

### 2.4. Chemical Analysis of AE

Total caffeoylquinic acids content of AE was determined by referring the method of Chinese Pharmacopeia. The absorption was measured in quartz well at 305 nm using a Uv-vis spectrophotometer assay (Shimadzu, Japan). The results were expressed as gram 1,3-dicaffeoylquinic acid equivalent/100 g AE. The calibration equation for 1,3-dicaffeoylquinic acid was *y* = 8.6942*x* + 0.02318 (*R*2 = 0.9992) within the concentration range of 10–120 *μ*g/mL.

**Figure 1:**
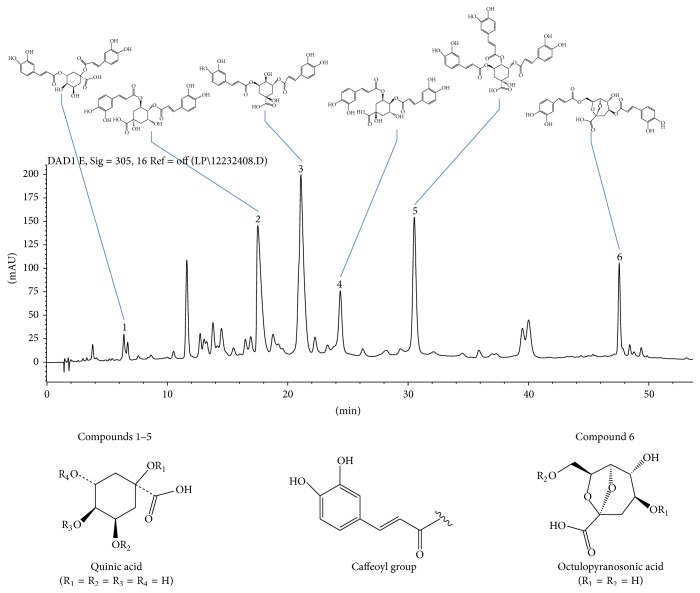
HPLC chromatogram of the caffeoylquinic acid derivatives.

HPLC analysis experiments were performed on an Agilent 1200 HPLC system (Agilent Technologies, USA) with diode array detector. An Agilent C_18_ column (150 mm × 4.6 mm, 5 *μ*m) was used to separate the compounds and the gradient elution was performed with a flow rate of 1.0 mL/min. The wavelength was set at 305 nm. The column temperature was set at 25°C. The mobile phase consisted of 0.1% aqueous acetic acid and acetonitrile. The sample injection volume was 10 *μ*L. The gradient elution program was as follows: 0–40 min, 13–25% acetonitrile; 40–50 min, 25–40% acetonitrile; 50–60 min, 40–50% acetonitrile. The content of total caffeoylquinic acids was 81.7% in AE by Uv-vis spectrophotometer assay. Six caffeoylquinic acid compounds were determined by HPLC (shown in Figure 1). The results of HPLC analysis demonstrated that the major constituents of AE were 1,3-dicaffeoylquinic acid (0.7%), 3,4-dicaffeoylquinic acid (3.8%), 3,5-dicaffeoylquinic acid (10.4%), 4,5-dicaffeoylquinic acid (2.1%), 3,4,5-tricaffeoylquinic acid (7.2%), and Erigoster B (2.7%), respectively (shown in Table 1).

### 2.5. Myocardial Ischemia Reperfusion Surgeries and AE Treatment

Experimental surgeries were performed according to previous description with our modifications [14]. In brief, rats were anesthetized with pentobarbital sodium (i.p., 70 mg/kg body weight) and underwent endotracheal intubation and mechanical ventilation. After thoracotomy, pericardium was incised and the left atrium appendage was elevated to expose the LAD coronary artery. A 6-0 silk suture was passed around the LAD coronary artery, and the ends of the suture were threaded through a small vinyl tube to form a snare. I/R was induced by LAD ligation for 30 min, confirmed visually* in situ* by the appearance of regional epicardial cyanosis and ST-segment elevation. Reperfusion was introduced by releasing the snare gently and chest was closed, and the rats were extubated after recovery. Sham group rats were subjected to the entire surgical procedures except that the 6-0 silk suture was not ligated. After ECG monitoring at the end of the reperfusion period (24 h), blood was collected from aorta immediately before sacrifice and hearts were excised for following assays. Serum was separated by centrifugation (4000 rpm, 5 min at 4°C) for LDH and CK-MB assay. The left ventricle was excised for infarct size measurement by TTC staining immediately (5 hearts per group) or left ventricular ischemic regions were dissected under stereoscope and frozen in liquid nitrogen before being stored at −80°C (5 hearts per group) or placed in a tube containing 10% formalin solution. Rats were random divided into six groups: (I) Sham group (given saline vehicle without I/R); (II) I/R group with saline alone; (III) I/R group with AE of 10 mg/kg; (IV) I/R group with AE of 20 mg/kg; (V) I/R group with AE of 40 mg/kg; (VI) I/R group with CAPE 5 mg/kg. The vehicle or drugs were administered 1 min before reperfusion via single bolus tail vein injection.

### 2.6. Determination of ST-Segment Depression and Heart Rate

At 24 h of reperfusion, rats were anesthetized with by i.p. injection of pentobarbital sodium (40 mg/kg). Electrocardiogram (ECG) in lead II was recorded through the needle electrodes attached to the limbs continuously for 15 min with an electrocardiograph (ASB240U, AOSHENG, China). The ST-segment depression and heart rate were calculated offline.

### 2.7. Measurement of Infarct Size

Heart infarct size was measured by TTC staining method as described previously [15]. Left ventricle was sliced into six 2-3 mm thick transverse sections and incubated in 1% TTC solution in PBS (pH 7.4) for 5 min at 37°C and then fixed in 10% formalin solution (pH 7.0) for 24 h. TTC stains viable tissue a deep red color, and nonstained tissue is presumed to be infarcted. The images of the slice were captured by a LEICA digital camera 480 and infarct area in each slice was measured by computed planimetry with an image analyzing program Image J. The infarct weight of each slice was calculated by multiplying the ratio of infarct area within total area by the slice weight. The infarct weight of each slice was summed to produce the total infarct weight of each left ventricle. Infarct size was calculated by dividing the infarct weight by the total weight of the left ventricle [16].

### 2.8. Western Blot Analysis

Western blot analyses of total proteins and phosphorylated form of proteins in hearts were performed according to methods described previously with modifications. Tissue in left ventricular ischemic regions was homogenized in ice-cold lysis buffer (sucrose 250 mM, Tris-HCl (pH7.2) 50 mM, sodium EDTA 2 mM, beta-mercaptoethanol 2 mM, sodium fluoride 5 mM, sodium orthovanadate 1 mM, aprotinin 10 *μ*g/mL, and leupeptin 10 *μ*g/mL) for 5 min. Equal amounts of protein were separated using 10% SDS polyacrylamide gel and transferred onto Immobilon-P membrane (pore size: 0.45 *μ*m, Millipore, USA). Rabbit polyclonal antibodies against phospho-JNK, total JNK, phospho-NF-*κ*B p65, total NF-*κ*B, p65 I*κ*B*α*, and *β*-actin were used to analyze expression levels of signaling molecules as described. Band intensities were quantified using a densitometer analysis system (Quantity One software, Bio-Rad).

### 2.9. ELISA for TNF-*α* and IL-6

Cardiac tissue levels of TNF-*α* and IL-6 were measured using ELISA kits for TNF-*α* (RayBiotech, Inc., Norcross GA) and IL-6 (R & D System). Frozen left ventricular tissues were homogenized using a homogenizer (IKA, Germany) in ice-cold PBS containing 0.05% sodium azide. Homogenates were sonicated for 15 seconds 3 times and centrifuged (3000 rpm, 10 min at 4°C) and resulting supernatants were used for ELISA determination. The contents of total protein in the supernatants were determined by using a Bio-Rad kit (Bio-Rad Laboratories, Hercules, CA, USA). Duplicate sample and standards were performed in each assay. Results were presented as pictograms per milligrams of total proteins.

### 2.10. Determination of Serum LDH and CK-MB Activities

LDH and CK-MB were two important markers of myocardial I/R injury. Activities of LDH and CK-MB in serum were determined by using commercial assay kits according to manufacturer's instructions and results were presented as units per liter.

### 2.11. Histological Evaluation of Leukocyte Infiltration

The heart tissue was fixed in 10% formalin at 4°C for at least 3 h and dissected into 7 *μ*m thick paraffin-embedded sections. The left ventricular paraffin sections were stained with hematoxylin and eosin (H&E). Leukocyte infiltrations were examined by light microscopy and counted at magnification ×20 (10 fields per heart specimens).

### 2.12. Determination of MPO Activity

MPO activity was determined by an MPO ELISA kit according to manufacturer's instruction. Frozen left ventricular tissues were homogenized in ice-cold PBS containing leupeptin (1 *μ*g/mL), pepstatin A (1 *μ*g/mL), and antipain (50 *μ*g/mL) and centrifuged, and the supernatants were collected for used for MPO activity assay. The results were calculated by a standard curve provided by the kit and presented as nanograms per milligram of left ventricular tissues.

### 2.13. Statistical Analysis

Data were expressed as mean ± SEM. Significance was determined by one-way analysis of ANOVA using SPSS 16.0 software (SPSS, Inc., Chicago, IL, USA). For single and multifactorial analysis,* post hoc* test(s) were performed to measure individual group differences of interest. Differences were considered significant if *P* < 0.05.

## 3. Results

### 3.1. AE Reduced Myocardial Infarct Size after I/R

**Figure 2:**
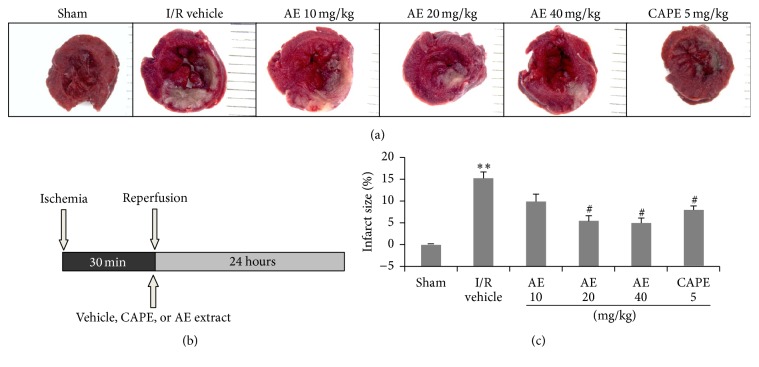
Effects of AE on myocardial infarct size after I/R in rats. (a) Representative TTC stained left ventricle slices are shown. Deep red-staining areas indicate normal tissue and unstained pale areas indicate infarct tissue. (b) Experimental protocol. Hearts were subjected to 30 min ischemia followed by 24-hour reperfusion. Different doses of AE (10, 20, or 40 mg/kg), CAPE 5 mg/kg, or saline (1 mL/kg) were administered via the tail vein 1 min before reperfusion. (c) Myocardial infarct size determined by TTC staining. *n* = 5/group. ^*∗∗*^ *P* < 0.01, versus Sham; ^#^ *P* < 0.05 versus I/R vehicle.

To evaluate the effect of AE against myocardial ischemia, we first determine whether AE was effective at ameliorating myocardial infarction after I/R. SD rats were subjected to 30 min ischemia followed by 24 h of reperfusion (Figure 2(b)) and infarct size was evaluated by TTC staining (Figure 2(a)). AE at 3 different dosages, CAPE (positive control), or saline (vehicle) was injected intravenously via the tail vein 1 min before reperfusion. The infarct sizes were significantly decreased in AE-treated rats at 20 and 40 mg/kg groups compared with the vehicle-treated group (5.4 ± 1.3% and 4.9 ± 1.1% versus 15.2 ± 1.4% vehicle, *P* < 0.05). The low dosage AE at 10 mg/kg also decreased the infarct size but different did not reach a statistical significance. Administration of CAPE afforded a significant reduction with the infarct size at 7.9 ± 0.9%. These results suggested that AE reduced myocardial infarct size after I/R injury in dose-response manner (Figure 2(c)).

### 3.2. AE Suppressed I/R-Induced ST-Segment Depression

**Figure 3:**
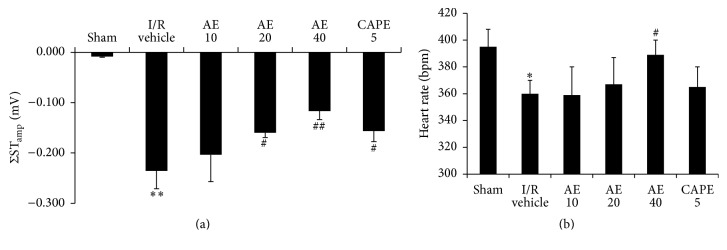
Effects of AE on ST-depression and heart rate of ECG after I/R in rats. (a) ST amplitude changes (ΣST_amp_). (b) The lead II ECG was recorded continuously for 15 min at 24 hours after I/R. Different doses of AE (10, 20, or 40 mg/kg), CAPE 5 mg/kg, or saline (1 mL/kg) were administered via the tail vein 1 min before reperfusion. *n* = 10/group. ^*∗*^ *P* < 0.05, ^*∗∗*^ *P* < 0.01, versus Sham; ^#^ *P* < 0.05, ^##^ *P* < 0.01 versus I/R vehicle.

The amplitude of ST-segment and heart rate were recorded and used as the index of ischemic electrocardiograph changes. As shown in Figure 3(a), a total mV of ST-depression (ΣST_amp_) was approximately 0.2 mV in the anesthetized rats after I/R, indicating a marked ischemia as compared with hearts with Sham operation. Administration of AE at 20 and 40 mg/kg significantly inhibited 24-hour reperfusion-induced ST-segment depression. CAPE (5 mg/kg) also showed significant protective effect against the I/R-induced ST-segment depression. Heart rate decreased after 24-hour reperfusion in vehicle-treated group, as compared to Sham group (Figure 3(b)). Similar to the case of ST-depression, AE at 40 mg/kg inhibited the heart rate decrease significantly. AE at 10 or 20 mg/kg had no effect, and CAPE had a slight effect without significant difference (*P* > 0.05). These results suggested that AE exerted potent protective effects on the ischemic electrocardiography changes induced by 30 min ischemia and 24-hour reperfusion in rat hearts.

### 3.3. AE Decreased I/R-Induced Myocardial Necrosis

**Figure 4:**
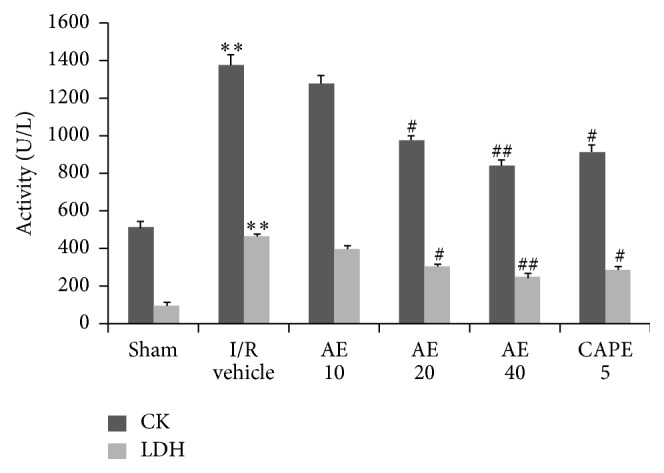
Effects of AE on CK and LDH activities in serum after myocardial I/R in rats. Hearts were subjected to 30 min ischemia followed by 24 hours' reperfusion. Different doses of AE (10, 20, or 40 mg/kg), CAPE 5 mg/kg, or saline (1 mL/kg) were administered via the tail vein 1 min before reperfusion. *n* = 5/group. ^*∗∗*^ *P* < 0.01, versus Sham; ^#^ *P* < 0.05, ^##^ *P* < 0.01 versus I/R vehicle.

The serum levels of both CK-MB and LDH, as two markers of myocardial necrosis, were significantly increased by I/R in vehicle-treated group (Figure 4). AE at 20 (975 ± 26.6 versus 1375 ± 56.8 U/L for CK-MB, *P* < 0.05; 306 ± 14.7 versus 465 ± 15.6 U/L for LDH, *P* < 0.05) or 40 mg/kg (841 ± 31.2 versus 1375 ± 56.8 U/L for CK-MB, *P* < 0.01; 246 ± 23.5 versus 465 ± 15.6 U/L for LDH, *P* < 0.01) generated a significant decrease in serum CK-MB and LDH levels, as well as by CAPE treatment (913 ± 40.2 for CK-MB, *P* < 0.01; 287 ± 18.8 for LDH, *P* < 0.01). AE at dose of 10 mg/kg did not exhibit significant activity in this assay compared to* t* vehicle-treated group.

### 3.4. AE Reduced Production of Proinflammatory Cytokines

**Figure 5:**
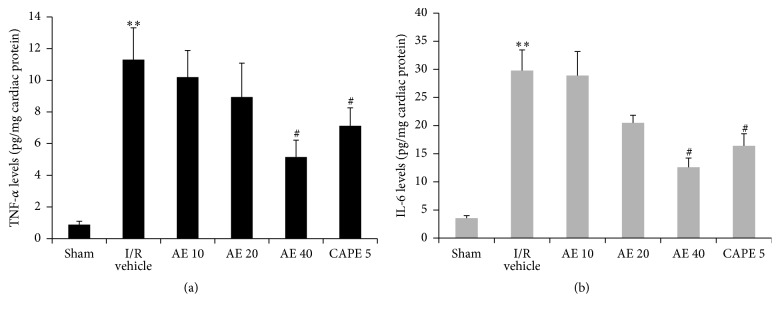
Effects of AE on TNF-*α* and IL-6 after myocardial I/R in cardiac tissue. Hearts were subjected to 30 min ischemia followed by 24-hour reperfusion. (a) TNF-*α*. (b) IL-6. Different doses of AE (10, 20, or 40 mg/kg), CAPE 5 mg/kg, or saline (1 mL/kg) were administered via the tail vein 1 min before reperfusion. *n* = 5/group. ^*∗∗*^ *P* < 0.01, versus Sham; ^#^ *P* < 0.05 versus I/R vehicle.

To determine the effect of AE on proinflammatory cytokines, cardiac tissue levels of TNF-*α* and IL-6 after I/R were evaluated. As shown in Figure 5, TNF-*α* and IL-6 levels were significantly elevated in vehicle-treated rats subjected to I/R injury. Administration of AE and CAPE markedly decreased levels of TNF-*α* and IL-6. AE at 40 mg/kg has significant effects on TNF-*α* and IL-6 levels, suggesting that cardioprotective effect of AE is associated with reduction of proinflammatory cytokines.

### 3.5. AE Inhibited the Activation of NF-*κ*B

**Figure 6:**
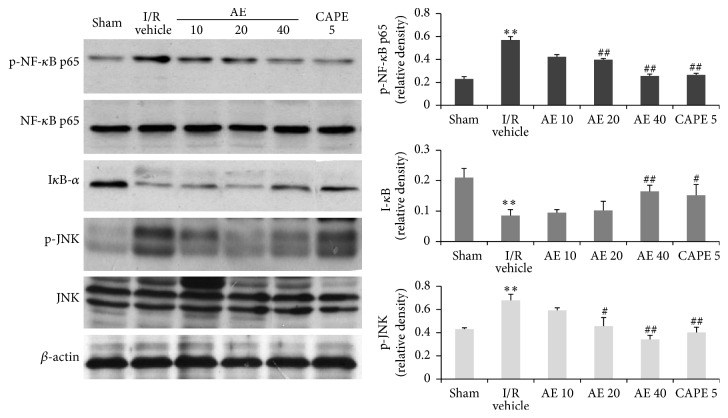
Effects of AE on phosphor-NF-*κ*B and phosphor-JNK expressions after I/R in rats. Phosphor-NF-*κ*b and phosphor-JNK were analyzed by western blot. Identical results were obtained in 5 separate rat hearts at each time point. Normalization of western blot was ensured by *β*-actin. Different doses of CA (5, 10, or 20 mg/kg), SA extract 40 mg/kg, or saline (1 mL/kg) were administered via the tail vein 1 min before reperfusion. ^*∗∗*^ *P* < 0.01, versus Sham; ^##^ *P* < 0.01, ^#^ *P* < 0.05 versus I/R vehicle.

To elucidate the effects of AE on NF-*κ*B pathway, phosphorylation of NF-*κ*B at Ser536 and expression level of I-*κ*B were detected in myocardium after I/R. Western blot analysis showed that I/R markedly upregulated NF-*κ*B phosphorylation (p-p65) and decreased I-*κ*B as compared with cardiac tissue of rats with Sham operation (Figure 6). AE treatment (20 and 40 mg/kg) inhibited the I/R-induced NF-*κ*B activation and I-*κ*B degradation. NF-*κ*B in myocardial tissue was identical with the effect of AE reduced myocardial ischemia reperfusion injury in rat, indicating AE inhibited NF-*κ*B activation to suppress inflammatory response and survival after myocardial infarction.

### 3.6. AE Attenuated the Phosphorylation of JNK

JNK was a stress-related kinase and its activation was induced by inflammatory stress in numerous types of cell, including cardiomyocytes. We further investigated the effect of AE on the occurrence of phosphorylated JNK. The western blot analysis revealed, that compared to Sham group, the expression of p-JNK from vehicle-treated group was increased significantly after I/R injury, while the protein level of total JNK showed no difference. Administration of AE (20 and 40 mg/kg) and CAPE before reperfusion markedly reduced JNK phosphorylation, suggesting attenuation of JNK subsequent translocation into the nucleus.

### 3.7. AE Inhibited Leukocyte Infiltration

**Figure 7:**
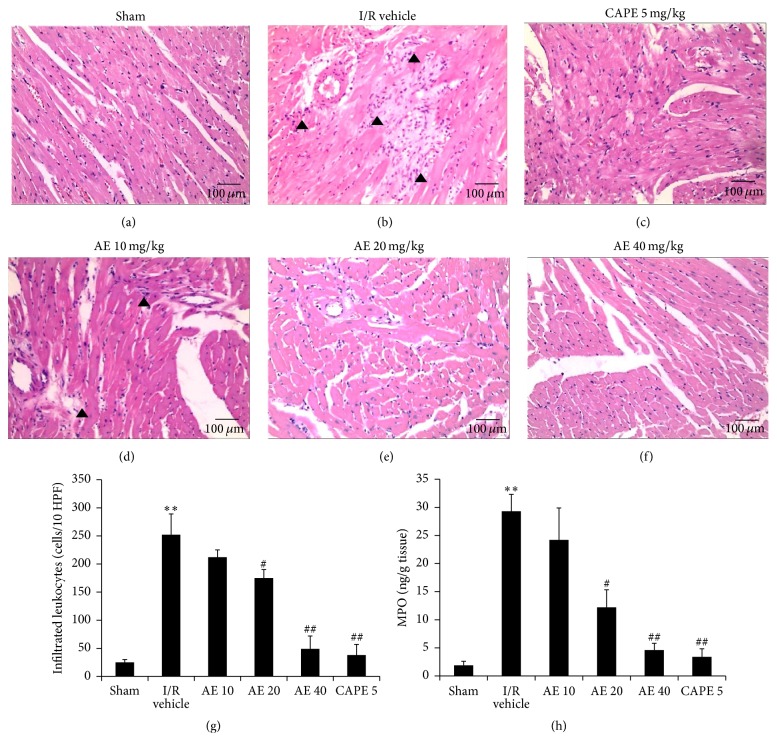
Effects of AE on histopathological changes after I/R in hearts. (a–f) Left ventricular sections stained with hematoxylin and eosin from representative rats are shown (×20, arrowheads, leukocytes infiltration). (a) Sham. (b–f) Hearts were subjected to 30 min ischemia followed by 24-hour reperfusion. Saline ((b), 1 mL/kg), CAPE 5 mg/kg 40 mg/kg (c), or different doses of AE (10, 20, or 40 mg/kg) were administered via the tail vein 1 min before reperfusion. (g) Number of infiltrated leukocytes, 10 fields per heart specimen. (h) MPO activities. ^*∗∗*^ *P* < 0.01, versus Sham; ^#^ *P* < 0.05 versus I/R vehicle; ^##^ *P* < 0.01. *n* = 5 in each group. Leucocyte infiltration ▲.

**Figure 8:**
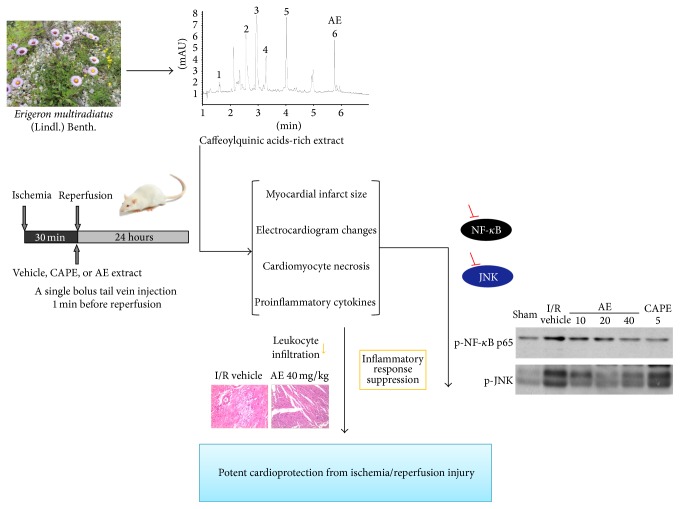
Graph chart of this study.

Histological analyses of hearts (Figures 7(a)–7(f)) demonstrated that I/R-induced widespread tissue necrosis in infarct area including leukocyte infiltration, interstitial hypercellularity, contraction bands, capillary compression, and abundant signs of hemorrhage (Figure 7(b)). AE treatment (10 mg/kg shown as in Figure 7(d); 20 mg/kg shown as in Figure 7(e)) reduced the above I/R-induced histopathological changes and hearts of AE-treated rats at 40 mg/kg (Figure 7(f)) appeared normal or there were only few interstitial edema. The decreased adherent and infiltrated PMNs (Figure 7(g)) in AE-treated groups reflected the anti-inflammatory activity of AE in the hearts after I/R. AE treatment at 20 and 40 mg/kg (Figure 7(h)) significantly inhibited MPO activity induced by I/R.

## 4. Discussion

In this study for the first time, we showed that a single bolus injection of caffeoylquinic acids-rich extract of* E. multiradiatus* (AE) alleviated acute myocardial ischemia reperfusion injury in rat. This was reflected by a reduction in infarct size, suppression of ST-depression, and decrease in CK-MB and LDH. These effects of AE correlated with inhibition of leukocyte infiltration and reduction of TNF-*α* and IL-6 production in the ischemic myocardial tissues. The cardioprotective effect of AE was associated with the suppressing I/R-induced inflammatory response, thereby inhibiting NF-*κ*B and JNK activation after reperfusion.

After myocardial infarction occurred, restoration of blood flow was the best effective way to save the myocardium from ischemic damage. Reperfusion exacerbated the myocardial injury though many pathological mechanisms, of which the burst of inflammatory response in ischemia cardiomyocyte was the mostly recognized cause. Turillazzi et al. evaluated detectable morphological changes in myocardial specimens of fatal MI patients and suggested essential markers (i.e., IL-15 and MCP-1) as early indicators of myocardial inflammatory response to MI. Extensive experimental work suggests that several inflammatory mediators have shown promise as therapeutic targets. Highly selective approaches to inhibit inflammation-driven protease activation and to promote recruitment of reparative cells may exert beneficial actions on the infarcted heart. However, anti-inflammatory strategies used in clinical trials may have been suboptimal. Saxena et al. discussed the most possible explanation of the translation failure of anti-inflammatory approaches, which reflect the limited role of inflammatory cardiomyocyte injury during the very early stages of MI [17]. After the demonstrated therapies to reduce ischemic injury, one important progress has been made in evaluating protective interventions against lethal reperfusion injury over the past decades [18].

Activation of cytokine cascades and oxidative stress after reperfusion in the infarcted myocardium was established in numerous studies [2]. Experimental studies in animals revealed that pharmacological treatments of phenolic acids might be an effective process for treating myocardial infarction. Unfortunately, despite demonstration of cardioprotection by phenolic acids intervention in studies* in vitro* or* in vivo*, translation of these effects into clinical application mostly remained unsuccessful. The most possible reason was that predicting when myocardial infarction occurred and preventing the infarction with long-time antioxidants phenolic acids use was impractical. Administration of pharmacological agents after myocardial infarction and just before the blood reflow might be a more effective way to treat myocardial I/R injury. In this study, we administered a single bolus of AE in normal saline at 3 doses (10, 20, and 40 mg/kg) intravenously to rats 1 min before reperfusion and evaluated the cardiac effect of AE on I/R injury.

Caffeoylquinic acids, including monocaffeoylquinic acids, dicaffeoylquinic acids, tricaffeoylquinic acids, and multicaffeoylquinic acids, were enormously reported both in phytochemical and in pharmacological aspects. Recently published data showed that caffeoylquinic acids exhibited significant activities such as antioxidation, anti-inflammation [19, 20], neuroprotective [16, 21], hepatoprotective [22], and cardioprotective [23] activities with minimum adverse effects. In this study, we investigated the protective effect of caffeoylquinic acid derivatives isolated from* E. multiradiatus* (AE) against I/R injury in heart, using the positive control CAPE, which was well-demonstrated to be a potent anti-inflammatory and antioxidant natural component. The interaction of these two main activities in addition to other biochemical and pathological mechanisms of caffeoylquinic acids made these associated with a possible reduced risk of cardiovascular diseases. Preclinical studies reported that LPS-induced cardiac stress in rats was improved by CAPE pretreatment through upregulating heme oxygenase-1 and downregulating TNF-*α* [24]. Chang et al. reported that CAPE reduced reperfusion-induced ventricular fibrillation through inhibition of Ca^2+^ and Na^+^ inward currents in isolated guinea-pig hearts [25]. Another study showed that CAPE treatment exhibited protection against gamma irradiation-induced cardiac-oxidative impairment via modulation free radical production [26]. Moreover, CAPE prevented lipid peroxidation and alleviated the heart injury triggered by I/R through significant reduction in MPO and Na^+^/K^+^ ATPase activity [27]. Finally, no clinical data was available to elucidate its therapeutic benefit for cardiovascular diseases. We compared the cardiac potential of AE at 3 doses (10, 20, and 40 mg/kg) with CAPE at 5 mg/kg on acute myocardial I/R injury in rat. Our data suggested that AE at 20 and 40 mg/kg had higher potency than CAPE on therapeutical effect on myocardial ischemia. These six caffeoylquinic acids, including 1,3-dicaffeoylquinic acid, 3,4-dicaffeoylquinic acid, 3,5-dicaffeoylquinic acid, 4,5-dicaffeoylquinic acid, 3,4,5-tricaffeoylquinic acid, and Erigoster B appeared to be the major and active compounds accounting for the cardioprotection of AE against I/R injury.

Till now, mechanisms underlying myocardial I/R injury were extensively studied but were still not clear. Its pathogenesis was demonstrated to include inflammation, endothelial dysfunction, mitochondrial damage, cardiomyocyte apoptosis and necrosis, and ROS. The production of cytokines played a key role in producing and developing of acute myocardial ischemia. Within hours after reflow to ischemic myocardium, cytokines were secreted locally by all nucleated cells in endothelial cell, smooth muscle cell, and cardiomyocyte. Furthermore, polymorph nuclear neutrophils (PMNs) combined the intercellular adhesion molecule-1 (ICM-1) and released many inflammatory mediators with catalytic action, which aggravated the reperfusion injury of myocardial cells by infiltrating of ischemic myocardium. During I/R, inflammatory cytokines might modulate myocardial survival by various mechanisms including stimulation of hypertrophy and fibrosis, impairment of myocardial, contractile function, induction of apoptosis and stimulation of genes involved in myocardial remodeling. Expression and release of proinflammatory cytokines such as TNF-*α* and IL-6 were commonly used biomarkers, which contributed to upregulation of cell-adhesion molecules, cardiac functional depression and myocardial damage [28]. Our data thus suggested that AE alleviated the severity of myocardial injury induced by I/R associated with reduced production of these two proinflammatory cytokines. After myocardial ischemia, NF-*κ*B was reported to play either cardioprotective or cardiotoxic actions. Misra et al. suggested NF-*κ*B protected cardiac myocyte against acute hypoxia and reperfusion injury [29]. However, prolonged activation of NF-*κ*B appeared to be cardiotoxic in heart failure by inducing signaling cascades that triggered chronic inflammation [30]. Moreover, inhibition of NF-*κ*B activation was demonstrated to be less susceptible to I/R [31]. We demonstrated that AE markedly downregulated NF-*κ*B phosphorylation (p-p65) after 24-hour reperfusion. Degradation of I-*κ*B was increased by I/R, and the reduction of I-*κ*B was associated with the occurrence of NF-*κ*B phosphorylation. Our data revealed that after 24-hour reperfusion, chronic activation of NF-*κ*B signaling induced prolonged inflammatory state resulting in increased cells death and progression toward myocardial damage. Given that the inflammatory response did not only cascade through signaling to NF-*κ*B, crosstalk among a network might be required for the cardioprotective mechanism of AE. c-Jun N-terminal (JNK) is an inflammatory stress-activated protein kinase, which was phosphorylated by inflammation stimuli. And subsequent translocation of JNK into the nucleus was demonstrated to be involved in regulation of expression of proinflammatory cytokines, including TNF-*α* and IL-6. This study suggested that AE attenuated the phosphorylation of JNK after I/R. Therefore, we demonstrated that AE exerted a potent cardioprotective effect against I/R injury by a mechanism that could be due to its anti-inflammatory activity. This was derived from the results that caffeoylquinic acids from* E. multiradiatus*, which was capable of suppressing the inflammatory response, inhibited NF-*κ*B activation and attenuated JNK phosphorylation after I/R injury. The observation that AE inhibited the leukocyte accumulation and infiltration further provided evidences that suppression of inflammatory substances in ischemic myocardium was required for cardioprotection of AE. Additional support for our hypothesis was from the observation that AE exerted a protect effect on ischemic ECG changes associated with reduction of inflammatory substances, which could have major detrimental consequences on cardiac function [32].

In conclusion, our study demonstrates that AE attenuates myocardial injury* in vivo*, by reducing infarct size, suppressing ST-depression, and decreasing cardiomyocyte necrosis. AE also inhibits leukocyte infiltration and decreased proinflammatory cytokines. The possible mechanism might be due to the inhibition of NF-*κ*B activation and attenuation of JNK phosphorylation. Through a combination of pharmacological and analytical chemical approaches, our results showed that the AE suppressed cardiac inflammatory response and alleviated myocardial I/R injury. Therefore, remarkable activities of caffeoylquinic acids from* Erigeron multiradiatus* indicated their potential in the discovery and development of new natural drugs for treating cardiac ischemia reperfusion injury.

## Figures and Tables

**Table 1:** 

Number	Compounds	R_1_	R_2_	R_3_	R_4_	Content in AE (%)	Concentration (*μ*M)/AE (10 *μ*g/mL)
1	1,3-Dicaffeoylquinic acid	Caffeoyl	Caffeoyl	H	H	0.7	0.54
2	3,4-Dicaffeoylquinic acid	H	Caffeoyl	Caffeoyl	H	3.8	2.94
3	3,5-Dicaffeoylquinic acid	H	Caffeoyl	H	Caffeoyl	10.4	8.05
4	4,5-Dicaffeoylquinic acid	H	H	Caffeoyl	Caffeoyl	2.1	1.6
5	3,4,5-Tricaffeoylquinic acid	H	Caffeoyl	Caffeoyl	Caffeoyl	7.2	4.2
6	Erigoster B	Caffeoyl	Caffeoyl	—	—	2.7	1.9
